# Postprandial Energy Metabolism in the Regulation of Body Weight: Is there a Mechanistic Role for Dietary Calcium?

**DOI:** 10.3390/nu2060586

**Published:** 2010-05-28

**Authors:** Mario J. Soares, Wendy L. Chan She-Ping-Delfos

**Affiliations:** 1 Curtin Health Innovation Research Institute, Program of Nutrition, School of Public Health, Curtin University of Technology, GPO Box U 1987, Perth WA 6845, Australia; 2 Curtin Health Innovation Research Institute, Curtin University of Technology, GPO Box U 1987, Perth WA 6845, Australia; Email: w.chanshepingdelfos@curtin.edu.au

**Keywords:** postprandial, calcium, thermogenesis, obesity, fat oxidation, fecal fat, lipolysis

## Abstract

There has been much interest in the mechanisms by which calcium may attenuate weight gain or accelerate body fat loss. This review focuses on postprandial energy metabolism and indicates that dietary calcium increases whole body fat oxidation after single and multiple meals. There is, as yet, no conclusive evidence for a greater diet induced thermogenesis, an increased lipolysis or suppression of key lipogenic enzyme systems. There is however convincing evidence that higher calcium intakes promote a modest energy loss through increased fecal fat excretion. Overall, there is a role for dietary calcium in human energy metabolism. Future studies need to define threshold intakes for metabolic and gastrointestinal outcomes.

## 1. Introduction

Body weight and body energy content remains quite stable in most adults for long periods of time; despite daily fluctuations in energy intake and energy expenditure. This requires the presence of regulatory processes able to match fuel supply to energy requirements. The maintenance of a physiological set point for body weight is complex and includes aminostatic and glucostatic controls of feeding, metabolic or nutrient partitioning, input from the sympathetic nervous system, signals from adipose tissue as well as additional behavioral influences. Genetics, the environment and psychosocial factors also impact on these regulatory processes [1,2,3]. That a substantial proportion of people in the world suffer from obesity argues for a sustained dysregulation in the processes that otherwise serve to keep the system in check. 

**Figure 1:**
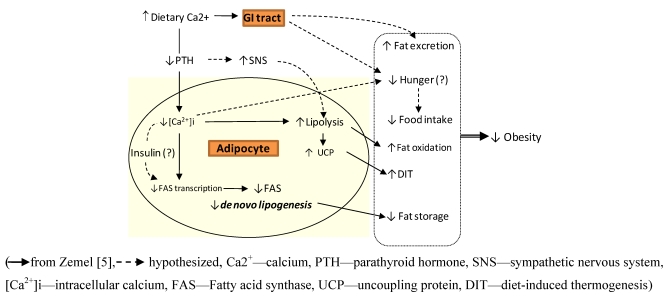
A calcium-body weight model describing potential anti-obesity effects of dietary calcium.

A role for calcium in the regulation of body weight has gained much interest since the early observation that increased intakes of calcium (as yoghurt) increased the loss of body fat and favoured a redistribution of fat away from the abdomen [4]. Based primarily on studies with the agouti mouse model, Zemel *et al.* [5] proposed that intracellular calcium (iCa^2+^) held the key to fat deposition and obesity. According to this early scheme (Figure 1) increases in dietary calcium would, via PTH, chronically lower iCa^2+^ in the adipocyte. Directly, or possibly via insulin, iCa^2+^ would then act to reciprocally regulate; a reduced expression of fatty acid synthase (FAS)—a key enzyme regulating lipid deposition—while stimulating adipose tissue lipolysis. An increased fat oxidation and thermogenesis through up-regulation of uncoupling proteins (UCP) was also suggested. 

We now know that dietary calcium may also act at the level of the gastrointestinal tract to increase energy loss, through increased fecal fat excretion (Figure 1). Tordoff [6] has hypothesized a ‘calcium appetite’ as an innate drive for calcium-rich food sources when faced with calcium depletion. This view highlights the importance of ascertaining links between calcium intake, subjective hunger/ satiety ratings and free living food intake. Whether such potential roles for calcium are mediated through the adipokine, leptin [7], or more locally via GI tract hormones also requires study (Figure 1). Other authors have hypothesized that the long term suppression of PTH may instead hold the key to obesity prevention [8]. A reduced activation of the sympathetic nervous system (SNS) has long been implicated in the weight gain of both animals and man. Chronic lowering of PTH would stimulate the SNS to allow greater thermogenesis and lipolysis. As argued by McCarty & Thomas [8], validation of such a pathway would be important to obesity management, since there are other approaches to suppressing PTH, besides increasing dietary calcium. 

## 2. Body Weight Regulation

In the main, energy balance is determined by the matching of macronutrient intake to energy expenditure and the ability to channel nutrients into oxidative versus storage pathways (nutrient partitioning). Most individuals reach a state of approximate weight maintenance in which the average composition of the fuels they oxidize matches the nutrient distribution in their diets [9]. Acute increases in protein and carbohydrate intake elicit powerful auto-regulatory increases in protein and carbohydrate oxidation respectively, leading to near zero balance. In contrast, the ability to stimulate fat oxidation to offset recent increases in fat intake is less accurate, and hence fat balance is more likely to be disrupted [9]. Hence fat balance, rather than the balance of other macronutrients, is equated to energy balance. The latter principle forms the cornerstone of obesity prevention programs whereby reductions in fat intake and increases in fat oxidation through voluntary physical activity, are advocated.

There are three main contributors to total energy expenditure (TEE) of man: basal metabolic rate (BMR) has the largest energetic demand accounting for 60–70% of TEE in sedentary individuals, diet induced thermogenesis (DIT) is a variable 10% and physical activity makes up the remainder. BMR is determined by fat free mass (FFM) to a great extent (80–85%) and by fat mass (FM) if the person is obese. Smaller contributions are made by age, habitual physical activity and genetics. DIT is traditionally calculated as the change in postprandial energy expenditure from fasting and expressed as a percentage of the energy content of the meal. DIT has two components: an obligatory process of energy expenditure that is strongly related to meal size, composition and the physiological characteristics of the individual, and a regulatory component that is strongly influenced by the SNS. A higher DIT for a given meal would imply that less energy is available for storage, and hence such a meal would be less conducive to weight gain. Abdominal adipocytes are particularly sensitive to SNS mediated lipolysis; a process whereby stored fat is broken down and mobilized. 

## 3. Calcium and Postprandial Energy Metabolism

This paper focuses on the potential postprandial events that would contribute to an anti-obesity effect of calcium based on the model in Figure 1. These would include: (i) an increased 24 h energy expenditure and thermogenesis, (ii) increased whole body fat oxidation, (iii) decreased fatty acid synthase expression and activity resulting in decreased de novo lipogenesis, (iv) increased lipolysis, and (v) increased energy loss from decreased fat absorption. There are only a few published human studies from 2,000 onwards that have employed a randomised design, and have conducted postprandial measurements that focus on these aspects [10,11,12,13,14,15,16,17,18,19,20]. These studies have been ordered on study duration as acute (<24h-1week), short term (weeks to 3 months) and long term (6 months or more) (Table 1). The subjects have been normal weight to obese, and authors have examined the effects of both dairy (D) and non-dairy (ND) calcium. There were variations in the formulations of ND calcium used, and in the absolute differences in calcium intake between control and experimental arms. These aspects would affect the consistency of final outcomes between studies designed for a particular purpose. 

Some methodological considerations are pertinent to the overall discussion on calcium and postprandial events. It is accepted that the previous evening’s meal composition affects the next day’s glycaemic response and macronutrient oxidation [21]. Since postprandial studies begin after an overnight fast, it would hence be necessary to control for any potential confounding across each participant on each occasion through standardization of the dinner meal. Further, differences in weight and volume of meal based challenges are known to affect gastric emptying, and hence postprandial metabolism. Differences in palatability of the test meals employed, is another consideration in study design. Sawaya *et al.* [22] observed that highly palatable meals acutely stimulated insulin and changed substrate utilization to favour carbohydrate oxidation. Ensuring that participants rate each test meal equally would be critical to making valid conclusions. Except for two [14,18], none of the other studies in this review provide any information on palatability of the different test meal employed. 

The absorption of calcium is not straight forward and is highly variable between subjects. There are many factors that may confound its absorption [23]. After standardisation of previous food intake (usually dinner) and test meal characteristics, two factors that deserve attention in study design are calcium bioavailability and the initial vitamin D status of the participant. As the calcium load increases the percent absorption declines exponentially, such that at very high single loads the percentage absorbed is very low [24]. Secondly even within the normal vitamin D range, there is a large variability in absorption making individuals at the lower end of the range more susceptible to poor absorption [25]. Single meal studies hence need to be more careful than dietary studies. In the latter, when 3–4 smaller doses are advocated to fulfil the prescribed intake, then net absorption of calcium over the day would be enhanced. We labour these points, because the ability of calcium to influence postprandial metabolism may well depend on the difference in absorbed and retained calcium between study diets, together with other methodological factors discussed above. On the other hand the non-absorption of calcium is not necessarily a negative outcome and may have other benefits within the GI tract.

### 3.1. Calcium, Fat Oxidation & Thermogenesis

The rise in insulin in response to mixed meal ingestion drives thermogenesis and promotes an increased carbohydrate oxidation rate (COR), with a suppression of fat oxidation rate (FOR). Circulating free fatty acids (FFA) and glycerol are also suppressed. Cummings *et al.* [14] tested whether increasing dietary calcium influenced DIT and FOR and whether this varied by source of calcium. In a single blind 3-way crossover design, overweight and obese subjects were given single breakfast meals that varied in both dairy (D, 532 mg) and non-dairy (ND, 575 mg) calcium. Relative to control (174 mg), FOR was significantly less suppressed following both higher sources. Some trend for a better effect of ND was seen but did not attain statistical significance. These data were accompanied by higher FFA and glycerol following both higher calcium intakes with no difference in glucose or insulin. It is accepted that variations in the glycaemic index of a meal have important metabolic effects on the next meal eaten in sequence [26,27]. The design commonly employed to study this phenomenon has manipulated breakfast meal composition and tested its effect on lunch [26,27,28]. We questioned whether calcium would have similar effects and conducted a crossover trial of two breakfast-lunch combinations in lean and obese subjects [18,29]. We observed that higher D calcium at breakfast (548 mg *vs.* 243 mg) modified postprandial FOR, DIT, FFA and glycerol with carry over effects to a standard lunch, low in D calcium (48 mg). Overall, there was a greater DIT and FOR after the higher breakfast-lunch trial [18,29]. This was accompanied by greater circulating FFAs and glycerol despite similar glycaemic and insulinaemic responses following these meals [30]. Together, these acute meal based studies provide some confirmation of the calcium-body weight model and suggest that calcium may not need to act via insulin, to change postprandial metabolism (Figure 1). Such second meal effects of calcium would auger well for dietary interventions where several meals are ingested over the day, as they would act to potentiate the effect of calcium in each subsequent meal. 

There are four crossover trials that manipulated calcium intake over a week and measured postprandial DIT and FOR (Table 1). Boon *et al.* [11] achieved intakes of ~1,260 mg/d from D and ND sources and compared them to a low calcium diet (349 mg/d). 24 h measurements of energy expenditure and FOR were made in a whole body calorimeter (WBC) on the 7th day. There was no effect on total 24 h energy expenditure or its components, *i.e.*, rest, sleep and exercise. Similarly postprandial 24 h FOR was not statistically different between treatments but a clear rank order (D > ND > control) was evident with a difference of ~8 g/d between high D and control diets. The authors calculated that such a difference could account for a loss of ~3 kg body weight if extrapolated to a year. Interestingly these trends for a greater 24 h FOR were paralleled by a similar trend for a greater negative fat balance across trials. In fact we note that in this study there was a significant negative fat balance following high D (−16.1 ± 6 g/24 h, paired t = 2.68, p < 0.05) but not after the other two. The study utilized high protein diets from either dairy or non-dairy sources, and also provided more dietary fibre than habitual consumed by the participants. Both components would be expected to influence calcium absorption and retention [31,32], but whether postprandial energy metabolism was affected is debatable at present. Overall the trends for greater 24 h FOR and more negative fat balance following high D offer rather encouraging data; especially since these were obtained during strict energy balance in a whole body calorimeter. 

Using a similar design, Jacobsen *et al.* [16] varied D calcium (1,800 mg/d) at two protein intakes (15%E *vs.* 23%E) for a week, and compared them to a control diet (protein 15%E, 500 mg/d). Whole body calorimetry (WBC) measurements at the start and end enabled the calculation of change over that week. There was a small but significant drop in weight across all 3 diets; accompanied by a significant drop in RQ. However, there were no differences in 24 h EE, energy balance or FOR. A closer examination of their results indicated that the change in 24 h COR adjusted for energy balance, favoured a greater suppression on the two high calcium diets. Reciprocally, the change in 24 h FOR showed a greater increase (~ 35%) on the high D (15%E protein) diet relative to control [16]. The small sample sizes would have precluded the detection of differences and is an issue that the authors have acknowledged [16]. In another study, normal and overweight subjects were studied twice on a low (~500 mg/d, 1 serve of D) and twice on a high calcium diet (~1,440 mg/d, 3 serves of D) [17]. On the 7th day of each trial (four trials in total), participants entered a WBC and were randomly subjected to conditions of 24 h energy balance or 24 h energy deficit (mainly through increased exercise). Higher D calcium increased 24 h FOR during conditions of energy deficit but not energy balance. This was ascribed to a greater FOR during exercise, rather than at rest or sleep, during the 24 h residency in the calorimeter. To the best of our knowledge this is the first demonstration of an augmentation of FOR during exercise by dietary calcium, and should auger well for future combined diet and exercise interventions for obesity. 

St-Onge *et al.* [19] studied 14 normal children with low calcium intakes. They were randomized to either a milk supplementation group (3 serves/d ~1,400 mg/d calcium) or a fruit beverage group (4 serves/d ~665 mg/d calcium) for a 1 week period, and after a washout were crossed over to the alternative treatment. Postprandial measurements were made at day 1 and on day 7 in response to a standard beverage of that treatment. BMR and DIT rose slightly, but did not change significantly on the milk treatment. In contrast the fruit group showed a significant fall in these two variables leading to significant phase x day interactive effects. Further RQ was lower and FOR higher after milk supplementation. It is of interest that the fruit flavoured beverage resulted in an intake of ~115 g/d of high fructose corn syrup (HFCS). We are unaware of studies relating changes in BMR, DIT or substrate oxidation to acute HFCS supplementation in humans. However the direction of outcomes obtained indicated a greater drop in BMR and DIT of the control group, rather an increase in the milk group. So the evidence for an effect of calcium is not direct. 

Ten obese men and women with calcium intakes <800 mg/d completed a double blind placebo controlled crossover study of 5 weeks. Calcium was provided as a milk mineral sachet twice a day to increase calcium by ~800 mg/d, over and above habitual intake. The control group received a placebo (malto-dextrin). This elegant study used tracer isotope technology, indirect calorimetry, micro-dialysis of sub-cutaneous fat and fat biopsies for gene expression. The authors reported no statistical difference in DIT or FOR over the 6h postprandial period [12]. In this protocol the calcium sachet or placebo was used as the meal challenge. The investigators also co-administered either caffeine or a placebo with the meal in a crossover design. Caffeine stimulated EE and FOR on the placebo arm of the study, but showed no difference on the high calcium diet [12]. One potential explanation may be that caffeine ingestion acutely suppressed calcium absorption from the sachet [33], and so nullified the expected interactive effect. In contrast, indirect calorimetry measurements in the postabsorptive (fasting) state without caffeine, showed that 5 week calcium supplementation increased FOR relative to the placebo arm by ~6.0 g/d. This difference was not significant, possibly due to the low power of the study for this effect. 

In a randomized parallel design, 3 groups of overweight and obese individuals were assigned to either control (~500 mg/d from 1 serve D plus placebo), high calcium (~500 mg/d from 1 serve D plus 900 mg/d calcium carbonate) and high calcium (~1,400 mg/d from 3 serves of dairy) during 12 weeks of energy restriction (~500 kcal/d). This was a sub-study of a multisite trial on the source of calcium and energy metabolism [20]. There was no difference in free living 24 h EE (from doubly labelled water) between treatments. DIT was significantly stimulated on the high D diet in response to a 30% saturated fat meal challenge, but was not significantly different from the other two. As with other studies in this area, the small sample sizes of each group (range 6–9 subjects) would have contributed to a poorer statistical power for between-group differences. However, fasting RQ was significantly lower on the ND diet. Further, change in RQ was also significantly lower on the ND (calcium carbonate) diet compared to no change on the control and a small rise on the D diet [20]. This resulted in a significantly higher estimated FOR on the ND compared to the other two. As RQs, but not COR, were reported it may be deduced that a higher COR following high D, resulted in the higher DIT on that diet. In contrast the higher FOR following the ND arm of the trial did not elevate DIT. When the data was examined independent of treatment allocation, there was a positive relationship between baseline 25(OH)D_3_ and change in DIT (r^2^ = 0.32, p = 0.004). The relationship of DIT to vitamin D status at baseline is interesting. It suggests that the ability of calcium to stimulate DIT is dependent on its absorption; a function of vitamin D status [25]. This may be particularly pertinent to this study, since all subjects were vitamin D deficient [20]. An alternative explanation is that 25(OH)D_3_ is linked to thermogenesis either directly or via PTH. Overall, ND calcium decreased RQ at rest and following a meal. 

There was only one long term randomized parallel study conducted over a year [15]. In this study, women with low calcium intake at the start were assigned to either a control (~673 mg/d) or high D calcium (~1057 mg/d) intervention. Postprandial responses to low and high D calcium test meals was measured at the start and after 12 months. The intervention group showed a greater increase in the DIT only following the low calcium test meal challenge. In contrast FOR was significantly greater in the intervention group following both the low and high calcium test meals. The data suggest that chronic intake of calcium changes the body's capacity for thermogenesis and fat oxidation, to promote FOR even following a low calcium meal. The greater FOR following the high calcium meal challenge in the high D group indicates that an acute on chronic effect was also evident. These authors also observed a significant inverse relationship between 1 yr changes in PTH and changes in fat oxidation following high calcium test meal. Such data provide would argue the case for PTH as the key modifiable variable in the calcium-body weight model (Figure 1). 

Dairy sources contain other bioactive components besides calcium and may be expected to show a greater effect. Dairy naturally contains vitamin D, which may be significantly higher in those countries that have mandatory fortification of their products. While vitamin D will enhance calcium absorption, there is some suggestion of an independent effect from the direct relation between 25(OH)D_3_ status and change in FOR [20] and the inverse association between ∆PTH and ∆FOR [15]. Further, supplementation with vitamin D improves insulin sensitivity [34,35] and by inference should modulate substrate oxidation. However, we are unaware of studies that have demonstrated such outcomes. In our single meal studies the higher D calcium meal had added vitamin D as well, but we observed only a trend for a better effect on FOR compared to ND [14]. A similar trend was also seen in the study of Boon *et al.* [11], however Teegarden *et al.* [20] found that the non-dairy (ND) group but not the dairy (D) group had higher FOR. Clearly, there is not enough data to make valid conclusions on the comparisons between sources of calcium. 

In summary this analysis shows that in 6 of 9 randomized studies that measured macronutrient oxidation, an increase in FOR following calcium was demonstrated (Table 1). We could discount the study of St-Onge *et al.* [19] for reasons discussed above. However, those of Boon *et al.* [11] and Jacobsen *et al.* [16] which showed strong trends, would need to be considered. Overall, given the range of subject, the small sample sizes, potential confounders in methodology and in calcium absorption, and the duration of studies (<24 h to 1 year), this is an impressive tally of positive studies. There is, however, little consistent evidence for an increase in DIT (Table 1) following higher calcium, in part due to a counter balancing by increased COR on the control or low calcium arm of these trials. An important consideration for future investigators would therefore be the determination of threshold intakes that promote both DIT and FOR from D and ND calcium sources, and the impact of different formulations of ND, on these processes. 

**Table 1:** Randomized controlled trials of higher calcium intake on postprandial energy metabolism.

Authors	Study diets & duration	Significance between treatments				
		↑DIT	↑FOR	↓Genes	↑Lipolysis	↑Fecal fat
Cummings, James & Soares, 2006 [14]	CO (n = 8). 3 breakfast meals 176 mg and 532mg (D) and 575 ND each over 6 hr.	No	Yes	--	Yes^1^	--
Soares *et al.*, 2004 [18]	CO (n = 11). 2 breakfast meals 248 mg and 543 mg (D), followed by standard lunch 48 mg (D) over 8 h.	Yes	Yes	--	Yes^1^	--
Boon *et al.*, 2005 [11]	CO (n = 12). 3 diets of 1259 mg/d (high D), 1259 mg/d (low D, calcium carbonate) and 349 mg/d calcium, each for 1 week.	No	No^2^	No	No	--
Jacobsen *et al.*, 2005 [16]	CO (n = 10). 3 dairy diets of 1,800 mg/d (15% protein), 1800 mg/d (23% protein) and 500 mg/d (15% protein), each for 1 week.	No	No	--	--	Yes^3^
Melanson *et al.*, 2005 [17]	CO (n = 19). Diets of 500 mg/d and 1,400 mg/d (D) each for 1 week and performed twice. Subjects studied on day 7 under energy deficit or in balance.	No	Yes^4^	--	--	--
St-Onge *et al.*, 2007 [19]	CO (n = 14). Milk or fruit flavoured sugar drink supplemented over 1 week each.	Yes	Yes^5^	--	--	--
Boon *et al.*, 2007 [10]	CO (n = 10). 4 diets of 2,500 mg/d, 1,200 mg/d and 400 mg/d from D and 1,200 mg/d from ND (calcium carbonate), each for 1 week.	--	--	Yes^6^	--	No^7^
Bortolotti *et al.*, 2008 [12]	CO (n = 10). D calcium (1,386 mg/d) or placebo (586 mg/d) over 5 weeks each.	No	No	No	No	--
Buchowski *et al.*, 2009 [13]	CO (n = 34). 500 mg/d and 1,500 mg/d calcium, each for 6 weeks weight loss, in lactose tolerant and intolerant subjects.	--	--	--	--	Yes
Teegarden *et al.*, 2008 [20]	PT (n = 24). 3 diets of 500 mg/d, 1400 mg/d (ND) or 1400 mg/d (D) each for 12 weeks of weight loss.	No	Yes	--	--	--
Gunther *et al.*, 2005 [36]	PT (n = 26). 2 diets of low (<800 mg/d) or high D (1,000-1,400mg/d) for 1 year.	No	Yes	--	--	--

CO = crossover trial, PT = parallel trial, DIT = diet induced thermogenesis, FOR = fat oxidation rate, Genes = genes relating to fatty acid synthase expression or those associated with fat metabolism. ^1^ indirect evidence through higher serum FFA and glycerol; ^2^ but significantly lesser fat balance on high D diet; ^3^ only on high D, 15% protein high calcium diet; ^4^ during caloric restriction, but not during energy balance; ^5^ mainly due to a greater drop in the alternative treatment;^6^ Decreased FAS and trend for greater HSL compared to habitual; ^7^ 56% higher on 2500 mg/d D diet, but not statistically different.

### 3.2. Calcium, Lipolysis and Lipogenesis

One expected effect of higher intakes of dietary calcium, is the reciprocal stimulation of lipolysis and suppression of de novo lipogenesis (Figure 1). Coppack *et al.* [37] and Frayn [38] have argued that following mixed meal ingestion, chylomicron triacylglycerol is preferentially acted upon by adipose tissue lipoprotein lipase. While re-esterification into triacylglycerol does occur, much of this FFA fails to be ‘trapped’ within adipose tissue and finds itself back in circulation. Raised FFA may then serve to drive fat oxidation. Does higher calcium intake somehow amplify this process and account for the acute responses seen with a meal challenge? In our studies with mixed meal challenges, increases in FFA, glycerol and FOR show a close parallelism following calcium [14,18,30]. We acknowledge that measurements of circulating glycerol are not the best index of lipolysis. The raised FFA and glycerol observed here and in studies by Zemel *et al.* [39,40] are only suggestive of greater lipolysis. Interestingly, Boon *et al.* [10] observed a trend (p = 0.078) of a greater inhibition of hormone sensitive lipase on a high calcium diet, providing some support for the model. Calcium dependant SNS activation has also been described [37]. A role for the SNS in postprandial events cannot be discounted as it contributes to meal-induced thermogenesis, and is a potent stimulator of adipose tissue lipolysis [37]. Recent evidence using abdominal subcutaneous microdialysis indicates that calcium supplementation (1,400 mg/d as milk mineral) for 5 weeks did not stimulate lipolysis, glycerol turnover or fat oxidation (Table 1) [12]. No differences in 24 h catecholamine excretion were also noted. 

We found only 3 human studies that have measured the mRNA expression of genes regulating fat metabolism, of which two were conducted by the same group. In the earlier study of Boon *et al.* [11], D and ND sources were used to increase calcium intake to ~1,300 mg/d but no difference in gene expression was noted. In the second study using the same design, duration and type of subject, Boon *et al.* [10] tested a greater range of calcium. While the 1,200 mg/d ND diet lowered plasma TAGs, the 2,500 mg/d ND diet decreased fat absorption and suppressed the mRNA expression of FAS. In contrast, Bortolloti *et al.* [12] studied obese subjects over 5 weeks and used 1,400 mg/d of D calcium but showed no difference in expression of genes for lipogenesis. The paucity of studies in the literature does not allow firm conclusions, but it would appear that at least 2,500 mg/d of ND calcium are required to demonstrate differences in gene expression. Clearly, more work is indicated in this challenging area. 

### 3.3. Calcium and Fat Excretion

Fecal fat excretion stems from exogenous (dietary) and endogenous sources (bile, fatty acids). Further, unabsorbed fat may be metabolised by bacteria in lower GI tract and hence not be excreted [41]. While not specific, the measurement of fecal fat serves to provide a snapshot of fatty acid absorption. Calcium may influence fat and energy metabolism by also forming insoluble calcium soaps with fatty acids. This may be particularly true for saturated fatty acids since they require a greater residence time in the GI tract for their absorption, and would be more available for the subsequent interaction with calcium. Calcium may also increase bile acid excretion and thereby decrease digestible energy of the diet. 

In Table 1, all the three studies that measured this endpoint showed an increase in fat excretion following calcium [10,13,16]. A key aspect of two of these studies was the interplay between calcium and protein on fat excretion. Jacobsen *et al.* [16] found a higher fat excretion on the 1,800 mg/d calcium diet with a normal (15%) protein content, but not on the 1,800 mg/d diet with 23% protein. A high protein intake is expected to enhance calcium absorption and so there may less luminal calcium available for interaction with fat. This may be particularly true for a high protein, low calcium combination (31). Boon *et al.* [10] favoured similar arguments for their observations, that on high protein intakes there were strong trends for greater fat excretion with greater calcium intake. In fact 2,400 mg/d of D calcium showed a 56% greater effect on fat excretion relative to the 400 mg/d diet but statistical significance was not achieved [10]. 

The study of calcium’s effect on fecal fat excretion predates the postulation of the calcium-body weight hypothesis, and there are many older studies that document increases in fecal fat excretion following higher calcium [41,42,43]. A recent meta-analysis confirms this outcome for both D and ND calcium. Christensen *et al.* (44) estimate that additional calcium may account for between 2–5 g/d greater loss of fecal fat. The effect of D calcium was more consistent than ND calcium, in part due to the many formulations of ND used. A regression analysis on a small number of subjects, predicted that ~1241 mg/d of D calcium would account for ~5.2 g/d of fat excretion. The authors estimate that such effects at best, would account for a 2.2 kg difference in body weight over 1 year [44]. Interestingly, these authors could not document a dose response relationship between these variables but allude to a possible threshold of ~800 mg/d for a calcium effect on fecal fat excretion. Besides the small effects on body weight, a lesser absorption of fat would have a dual benefit in the attenuation of postprandial TAGs, LDL-C and other lipid indices of cardiovascular risk. The latter is an area of much interest [45,46] but is not covered by this review.

## 4. Conclusions

It is our opinion that calcium modulates human energy metabolism. There is convincing evidence that calcium increases postprandial fat oxidation after a single meal or over a day. There is some suggestion that fasting FOR may be greater on a high calcium diet. However a higher DIT is not always observed. There is convincing evidence for calcium to modestly increase the amount of fat excreted, and thereby contribute to energy loss. The effect of dietary calcium on lipolysis and/or lipogenesis is inconsistent at present, and is a fertile area for future research. Nutrition and public health recommendations demand consistent observations with a strong mechanistic basis. Defining threshold effects for metabolic versus gastrointestinal function based on randomized controlled trials with adequate power, is one way forward. 
